# Supplementary material for: Mitotic kinase oscillation governs the latching of cell cycle switches
Source: Curr Biol. 2022 Jun 20;32(12):2780–2785.e2. doi: 10.1016/j.cub.2022.04.016 (PMC9616797; doi:10.1016/j.cub.2022.04.016)
Supplement: Document S1. Figures S1–S4 and Table S1 [file mmc1.pdf]

Current Biology, Volume *32*

Supplemental Information

Mitotic kinase oscillation governs the latching of cell cycle switches

Bela Novak and John J. Tyson

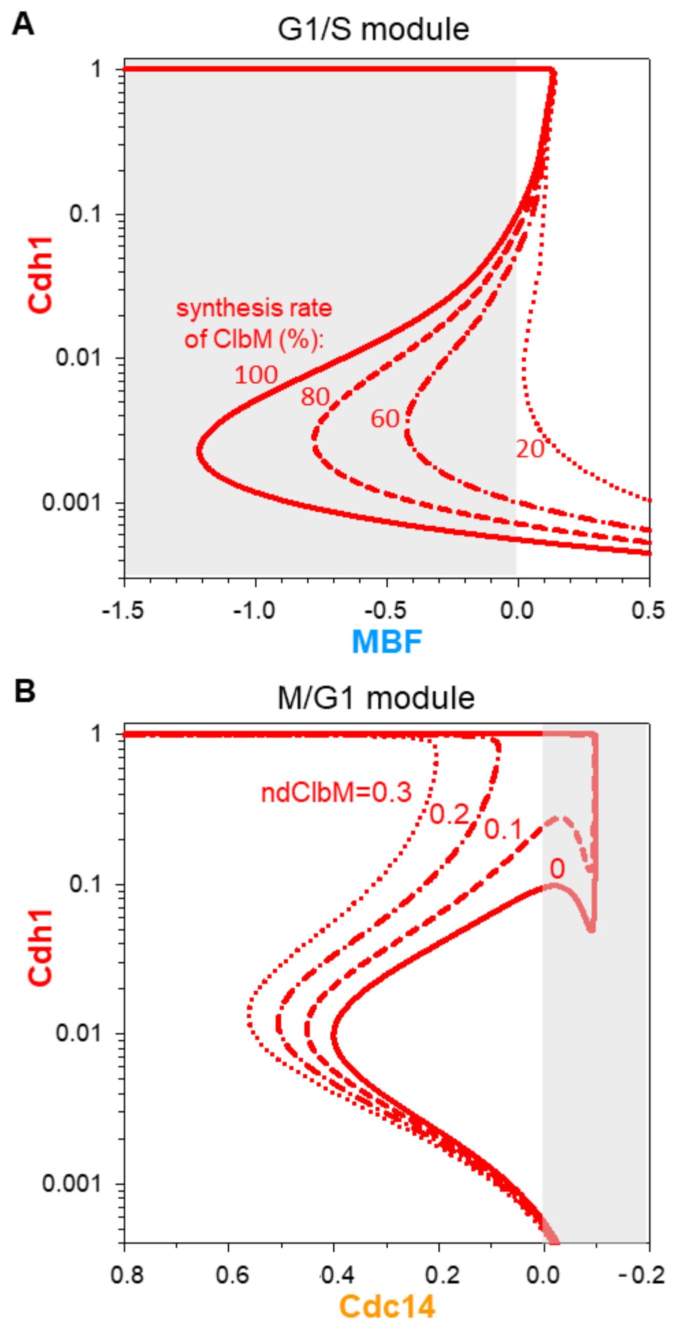

Figure S1. Bifurcation diagram for the G1/S and M/G1 modules. Related to Figure 3. (A) The steady-state level of Cdh1 as a function of MBF (when Cdc14=0) is Z-shaped, but the lower kink of the 'Z' lives in negative territory of MBF, so the switch latches at Cdh1≈0.0005 as MBF falls to zero. As the synthesis rate of ClbM decreases from its wild-type value (100%), the lower kink moves to larger values of MBF and eventually enters positive territory. Now the switch can flip back to Cdh1≈1 as MBF falls. **(B)** Similarly, the steady-state level of Cdh1 as a function of Cdc14 (when MBF=0) is Z-shaped, with the upper kink at a negative value of Cdc14. As the level of ndClbM increases from zero, the upper kink moves to larger values of Cdc14, eventually entering positive territory, and the switch fails to latch.

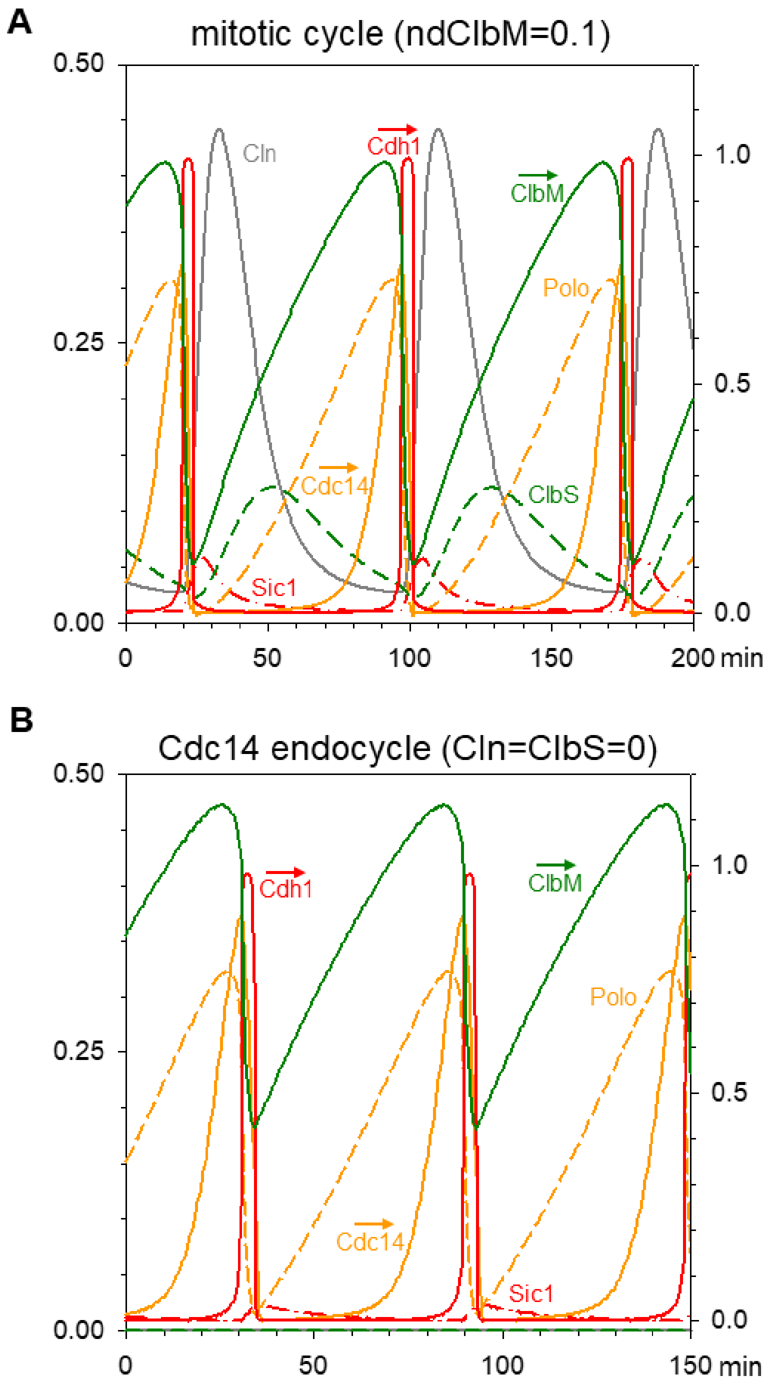

Figure S2. Properties of Cdc14 endocycles. Related to Figure 4. (A) Endocycles require a sufficiently high level of non-degradable ClbM. For ndClbM=0.1, the large post-mitotic peak of Cln, whose synthesis by SBF is very sensitive to inhibition by ClbM, indicates that ClbM activity must be very low (close to 0) as these cells exit mitosis. Indeed, the non-degradable fraction of ClbM is inhibited by the peak of Sic1 in early G_1. Low ClbM activity allows re-licensing of chromosome replication origins and the subsequent rise of ClbS activity is sufficient to initiate DNA replication and drive viable cell cycles. **(B)** Endocycles are independent of G_1- and S-phase cyclins (Cln=0 and Clbs=0).

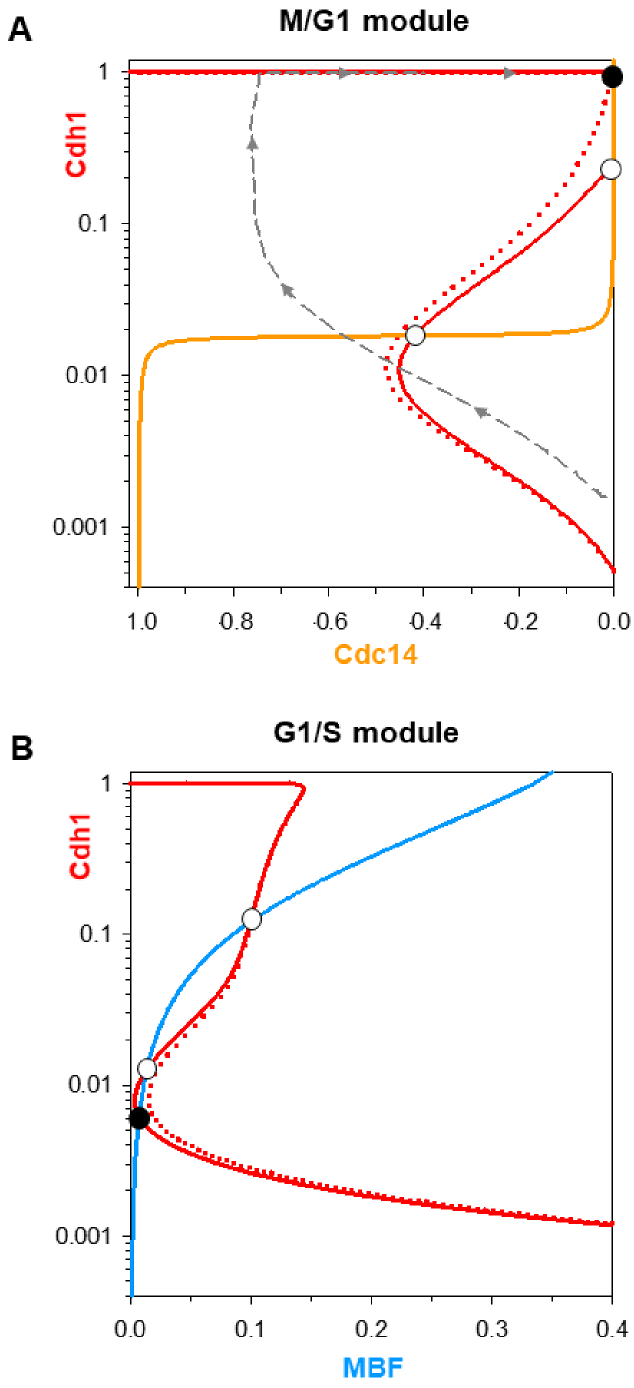

Figure S3. Endocycles arise by SNIPER ('saddle-node infinite-period') bifurcations. Related to Figure 4. Pseudo-phase plane for Cdc14 endocycles **(A)** and endoreplication cycles **(B)**, respectively. The red curves plot the locus of Cdh1 steady states for parameter values just below (solid) and just above (dotted) the SNIPER bifurcation points. As the parameter passes through the bifurcation point, a stable node (●) coalesces with an unstable saddle point (○), giving way to a limit cycle oscillation of 'infinite period'. For the solid and dotted red curves ndClbM=0.1 and 0.15 (A) and the rate of ClbM synthesis is reduced to 21% and 22.5% (B).

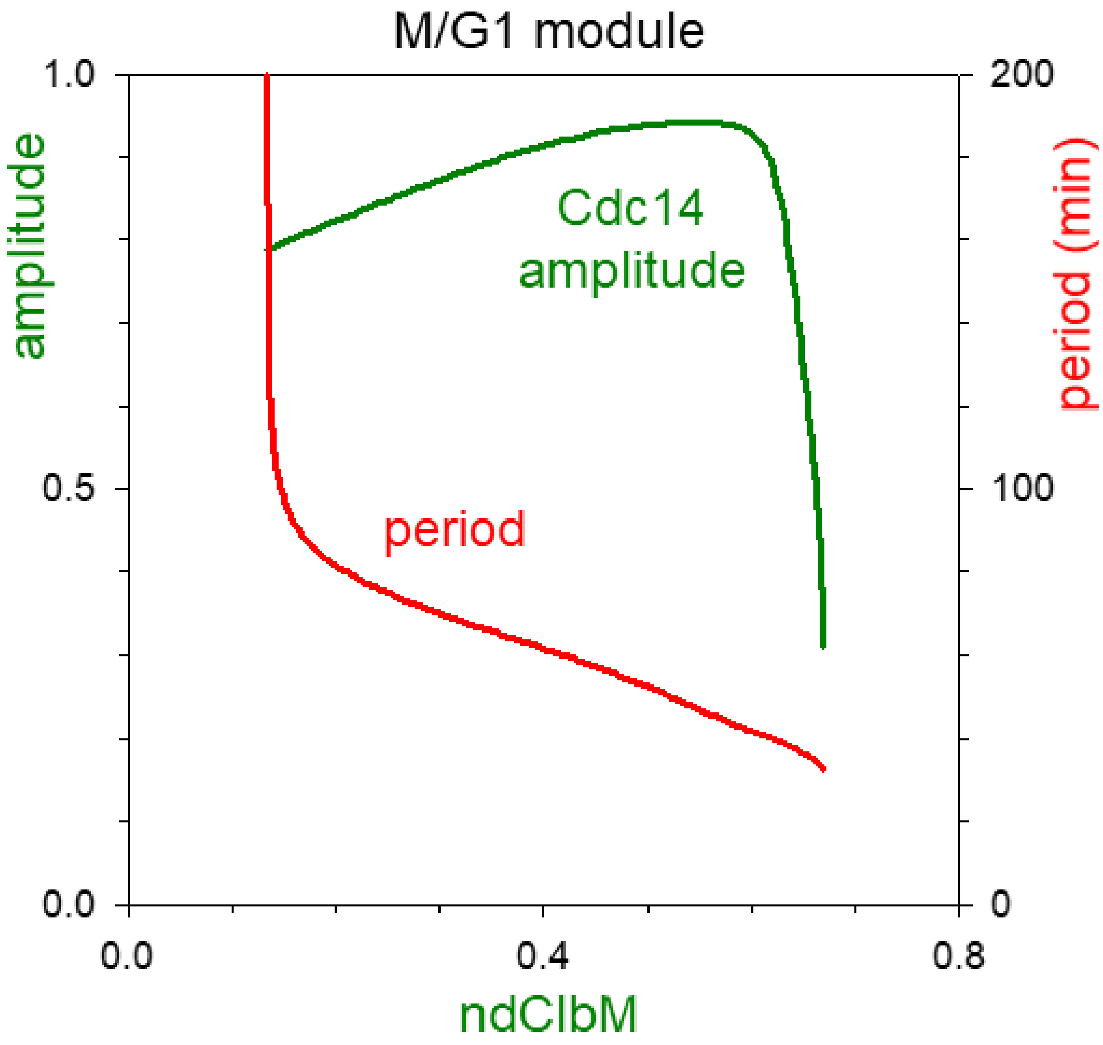

Figure S4. Bifurcation diagram for Cdc14 endocycles. Related to Figure 4. We plot the period and amplitude of the limit cycle oscillations as a function of the parameter ndClbM. Notice how the period diverges to infinity as ndClbM approaches the bifurcation point (ndClbM≈0.135).

Table S1. Parameter values of the budding yeast cell cycle model. Related to Star Methods.

**Cln synthesis/degradation & SBF regulation:**
$k_{scln} = 0.2$, $k_{dcln} = 0.2$, $k'_{asbf} = 1$, $k_{asbf} = 10$, $k_{isbf} = 25$, $J_{sbf} = 1$
**ClbS synthesis and degradation:**
$k_{sclbs} = 0.15$, $k'_{dclbs} = 0.1$, $k_{dclbs} = 0.05$
**MBF regulation by Nrm1:**
$k_{snrm1} = 0.05$, $k_{dnrm1} = 0.1$, $MBF_{tot} = 0.5$, k'_{ass}=1, k'_{diss}=0.001, $J_{mbf} = 0.01$
**ClbM synthesis and degradation:**
$k'_{sclbm} = 0.01$, $k_{sclbm} = 0.01$, $k'_{dclbm} = 0.01$, $k_{dclbm} = 1$, $J_{clbm} = 0.01$, $n = 2$
**Polo synthesis/degradation & Cdc14 regulation:**
$k_{spolo} = 0.01$, $k'_{dpolo} = 0.01$, $k_{dpolo} = 1$, $k_{acdc14} = 1$, $k_{icdc14} = 0.25$, $J_{cdc14} = 0.01$
**Sic1 synthesis/degradation & Clb binding :**
$k_{ssic} = 0.02$, $k'_{dsic} = 0.01$, $k_{dsic} = 2$, $J_{sic1} = 0.01$ $K_{diss} = 0.05$
**Cdh1 activation & inactivation:**
$k'_{acdh1} = 1$, $k_{acdh1} = 10$, $k'_{icdh1} = 0.2$, $k_{icdh1} = 10$, $J_{cdh1} = 0.01$
**Nondegradable ClbM:**
ndClbM=0 for mitotic and endoreplication cycle and ndclbM >0 for Cdc14 endocycle.
