# Supplementary material for: Mitotic kinase oscillation governs the latching of cell cycle switches
Source: Curr Biol. 2022 Jun 20;32(12):2780–2785.e2. doi: 10.1016/j.cub.2022.04.016 (PMC9616797; doi:10.1016/j.cub.2022.04.016)
Supplement: Methods S1. XPPAUT code for simulation of the budding yeast cell cycle model, related to STAR Methods [file mmc2.pdf]

XPPAUT code for simulation of the budding yeast cell cycle model. Related to Star Methods.

```
# XPPAut model for cell cycle latches
# Differential equations
Cln' = kscln*SBF - kdcln*Cln
ClbSt' = ksclbs*MBFa - (kdclbs' + kdclbs*Cdh1)*ClbSt
MBF' = kdiss'*(MBFtot - MBF) - kass'*MBF*(Nrm1t - (MBFtot - MBF))
Nrm1t' = ksnrm1*MBFa - kdnrm1*Cdh1*Nrm1t
ClbMt' = ksclbm' + ksclbm*ClbM^n/(Jclbm^n + ClbM^n) - (kdclbm' + kdclbm*Cdh1)*ClbMt
Polo' = kspolo*ClbM - (kdpolo' + kdpolo*Cdh1)*Polo
Sic1t' = kssic' - (kdsic' + kdsic*Clb*(Cln+Clb)/(Jsic1+Cln+Clb))*Sic1t
SBF' = (kasbf' + kasbf*Cln)*(1-SBF)/(Jsbf + 1 - SBF) - kisbf*ClbM*SBF/(Jsbf + SBF)
Cdh1' = (kacdh1' + kacdh1*Cdc14)*(1 - Cdh1)/(Jcdh1 + 1 - Cdh1) - (kicdh1'*Cln +
kicdh1*Clb)*Cdh1/(Jcdh1 + Cdh1)
Cdc14' = kacdc14*Polo*(1 - Cdc14)/(Jcdc14 + 1 - Cdc14) - kicdc14*Cdc14/(Jcdc14 + Cdc14)

# Algebraic equations for Clb & Sic1 binding
MBFa = MBF*Cln/(Jmbf + Cln)
Clbt = ClbSt + ClbMt + ndClbM
BB = Sic1t + Clbt + Kdiss
Sic1Clb = 2*Sic1t*Clbt/(BB + sqrt(BB^2 -4*Sic1t*Clbt))
Clb = Clbt - Sic1Clb
ClbM = (ClbMt+ndClbM)*(Clbt - Sic1Clb)/Clbt

# Auxiliary variables
aux ClbS = ClbSt*(Clbt - Sic1Clb)/Clbt
aux ClbM = (ClbMt+ndClbM)*(Clbt - Sic1Clb)/Clbt

# Parameter values
p kscln=0.2, kdcln=0.2, kasbf'=1, kasbf=10, kisbf=25, Jsbf=1
p ksclbs=0.15, kdclbs'=0.1, kdclbs=0.05
p ksnrm1=0.05, kdnrm1=0.1, MBFtot=0.5, kass'=1, kdiss'=0.001, Jmbf=0.01
p ksclbm'=0.01, ksclbm=0.01, kdclbm'=0.01, kdclbm=1, Jclbm=0.05, n=2
p kspolo=0.01, kdpolo'=0.01, kdpolo=1, kacdc14=1, kicdc14=0.25, Jcdc14=0.01
p kssic'=0.02, kdsic'=0.01, kdsic=2, Jsic1=0.01, Kdiss=0.05
p kacdh1'=1, kacdh1=10, kicdh1'=0.2, kicdh1=10, Jcdh1=0.01
p ndClbM=0

# XPP instructions
@ METH=stiff, XLO=0, XHI=250, YLO=0, YHI=1, total=250, dt=0.25, XP=time
@ NPLOT=8,YP=Cln,YP2=Cdh1,YP3=Sic1t,YP4=Nrm1t,YP5=Polo,YP6=Cdc14,YP7=ClbS,YP8=ClbM
done
```
