# Supplementary material for: Mitotic kinase oscillation governs the latching of cell cycle switches
Source: Curr Biol. 2022 Jun 20;32(12):2780–2785.e2. doi: 10.1016/j.cub.2022.04.016 (PMC9616797; doi:10.1016/j.cub.2022.04.016)
Supplement: Document S2. Article plus supplemental information [file mmc3.pdf]

Current Biology

Report

Mitotic kinase oscillation governs the latching of cell cycle switches

Graphical abstract

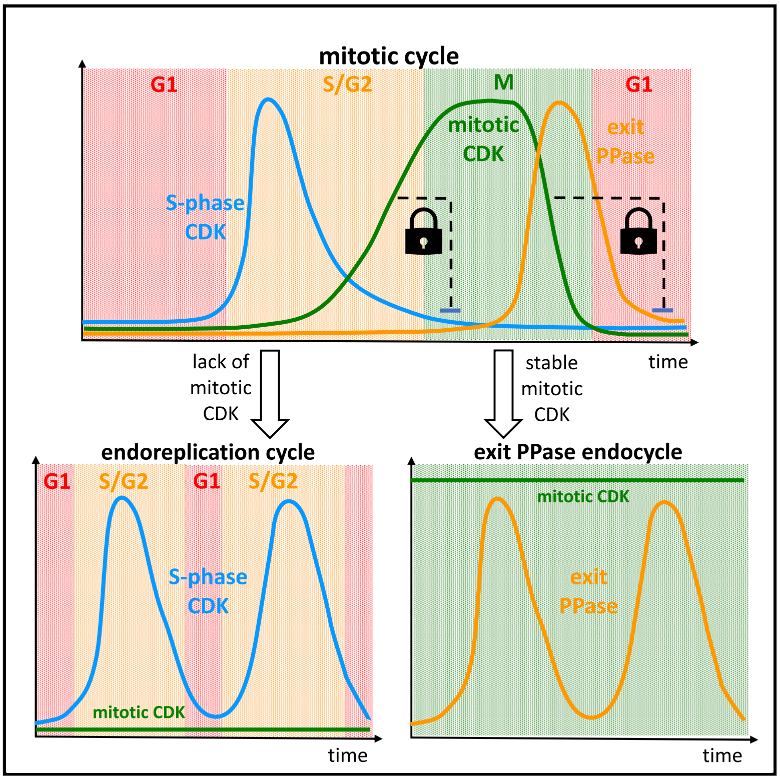

Highlights

- Mitotic kinases enforce once-per-cycle occurrence of cell cycle events
- Many cell cycle events can occur periodically in the absence of mitosis
- Are they autonomous oscillators or accidental consequences of faulty regulation?
- Regulatory mutations allow negative feedback loops to sustain oscillations

Authors

Bela Novak, John J. Tyson

Correspondence

bela.novak@bioch.ox.ac.uk (B.N.),
tyson@vt.edu (J.J.T.)

In brief

Periodic cell cycle events could be coordinated by an entrainment or a suppression mechanism. Using a mathematical model of the yeast cell cycle, Novak and Tyson show that autonomous oscillations of S-phase kinase and mitotic exit-phosphatase activities are suppressed by a "latching-gate" mechanism imposed by a universal mitotic Cdk oscillator.

Novak & Tyson, 2022, Current Biology *32*, 2780–2785
June 20, 2022 © 2022 The Author(s). Published by Elsevier Inc.
https://doi.org/10.1016/j.cub.2022.04.016

CellPress

CellPress
OPEN ACCESS

Current Biology

Report

Mitotic kinase oscillation governs the latching of cell cycle switches

Bela Novak[1,3,*] and John J. Tyson[2,*]
[1]Department of Biochemistry, University of Oxford, South Parks Road, Oxford OX1 3QU, UK
[2]Department of Biological Sciences, Virginia Tech, Blacksburg, VA 24061, USA
[3]Lead contact
*Correspondence: bela.novak@bioch.ox.ac.uk (B.N.), tyson@vt.edu (J.J.T.)
https://doi.org/10.1016/j.cub.2022.04.016

SUMMARY

In 1996, Kim Nasmyth[1] proposed that the eukaryotic cell cycle is an alternating sequence of transitions from G_1 to S-G_2-M and back again. These two phases correlate to high activity of cyclin-dependent kinases (CDKs) that trigger S-G_2-M events and CDK antagonists that stabilize G_1 phase. We associated these "alternative phases" with the coexistence of two stable steady states of the biochemical reactions among CDKs and their antagonists.[2,3] Transitions between these steady states (G_1-to-S and M-to-G_1) are driven by "helper" proteins. The fact that the transitions are irreversible is guaranteed by a "latching" property of the molecular switches, as we have argued in previous publications.[4,5] Here, we show that if the latch is broken, then the biochemical reactions can swing back-and-forth across the transitions; either G_1-S-G_1-S … (periodic DNA replication without mitosis or cell division) or M-(G_1)-M-(G_1) … (periodic Cdc14 release, without fully exiting mitosis). Using mathematical modeling of the molecular control circuit in budding yeast, we provide a fresh account of aberrant cell cycles in mutant strains: endoreplication in the *clb1-5*Δ strain[6] and periodic release and resequestration of Cdc14 (an "exit" phosphatase) in the *CLB2kd*Δ strain.[7,8] In our opinion, these "endocycles" are not autonomous oscillatory modules that must be entrained by the CDK oscillator[6,7] but rather inadvertent and deleterious oscillations that are normally suppressed by the CDK latching-gate mechanism.[8]

RESULTS AND DISCUSSION

As we have argued elsewhere,[2,4,5] cell proliferation (growth, DNA replication, mitosis, and cell division) is an alternating sequence of "one-way" transitions from G_1 (unreplicated chromosomes) into S-G_2-M (replication and partitioning of chromosomes) and back to G_1, which we picture dynamically (Figure 1A) as a series of flips of a bistable switch between two provisionally stable steady states, G_1 and S-G_2-M. Like a hinged gate with a latch in the "middle" position, the gate must be pushed sufficiently in one direction (by transcription factor, abbreviated as TF) to enter S-G_2-M, after which the gate swings back into the latched position. To return to G_1, the gate must be pushed sufficiently in the opposite direction (by exit protein, abbreviated as EP), after which it latches again. This latch has the unusual property that the gate must alternate between letting cells enter S-G_2-M and letting them leave. The latch catches because of the antagonistic interactions between cyclin-dependent kinases (CDKs) and their antagonists (the ubiquitin ligases and stoichiometric inhibitors that oppose S phase-promoting and M phase-promoting CDKs; Figure 1B). For the gate to latch properly, the mitotic cyclins must oscillate between low activity (G_1) and high activity (S-G_2-M), and the inducers (TF and EP) must be under negative-feedback control (i.e., TF must be inactivated after the cell enters S-G_2-M, and EP must be inactivated after the cell returns to G_1).

The G_1-to-S transition (activation of TF) and the M-to-G_1 transition (activation of EP) are controlled by "checkpoints" called Start (in yeast) and the spindle assembly checkpoint (SAC), respectively. To pass Start, a yeast cell must grow to a sufficiently large size, and to pass SAC, it must properly align its replicated chromosomes on the mitotic spindle. The mechanisms of these checkpoints have been described elsewhere.[9]

Our concept of the cell cycle as a latching gate has been verified by elegant experiments with budding yeast.[10,11] In this paper, we explore how perturbing the latch mechanism by mutation can convert a latching gate (Figure 2A) into a swinging gate (Figure 2B). In many cell types, deletion of mitotic cyclins may induce repeated rounds of DNA replication without mitosis ("endoreplication"[12]). On the other hand, if a nondegradable mitotic cyclin is expressed in budding yeast (so that a cell cannot properly exit mitosis), then cells exhibit sustained oscillations of a mitotic-EP (Cdc14 phosphatase; hence, "Cdc14 endocycles"[7,8]). How do these perturbations generate endocycles?

Mathematical model

To answer this question, we investigate a simple model of the budding yeast cell cycle (Figure 1C). At the heart of the network are double-negative interactions between ClbS and ClbM and their inhibitory substrates, Cdh1 and Sic1.[13] ClbS is regulated by two negative feedback loops involving its TF, MBF; namely MBF → Nrm1 –I MBF and MBF → ClbS –I Cdh1 –I Nrm1 –I MBF. ClbM is regulated by the negative feedback loop ClbM → Polo → Cdc14 → Cdh1 –I ClbM, and Cdc14 participates in its own negative feedback loop Cdc14 → Cdh1 –I Polo → Cdc14.

This is an open access article under the CC BY license (http://creativecommons.org/licenses/by/4.0/).

CellPress
OPEN ACCESS

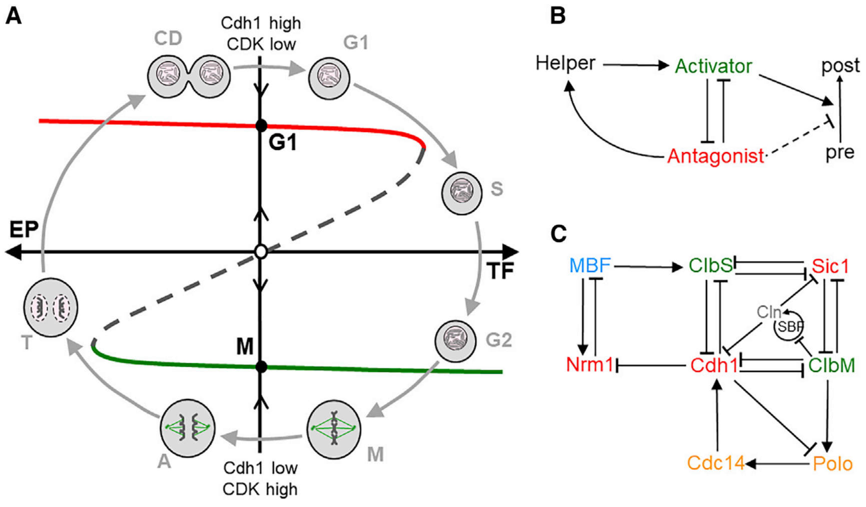

Figure 1. A "latching gate" model of the eukaryotic cell division cycle

(A) The protein interaction network controlling cell cycle progression is characterized by two coexisting, stable steady states (●): S-G_2-M with high activity of cyclin-dependent kinases (CDKs) and G_1 with high activity of CDK-antagonists (e.g., Cdh1), separated by an unstable steady state (○). TFs (transcription factors) and EPs (exit proteins) help to switch the control system from G_1 to S-G_2-M and from M to G_1. Red (green) line, locus of stable G_1-like (M-like) steady states; dashed line, locus of unstable steady states.

(B) Generic motif (barbed arrows indicate "activation" and blunt arrows "inhibition"). Mutual inhibition between activator and antagonist creates a bistable switch. The "helper" protein joins in by a negative feedback loop.

(C) A simplified protein interaction network for cell cycle control in budding yeast. ClbS (Cdc28:Clb6) and ClbM (Cdc28:Clb1–4) drive S and M phases, respectively; Cdh1 (APC/C:Cdh1) and Sic1 are antagonists of ClbS and ClbM; MBF (Mbp1:Swi6) is a transcription factor for ClbS; Cdc14 is a phosphatase that activates Cdh1; Nrm1 is a repressor of MBF-regulated gene expression; Polo is a kinase that promotes Cdc14 activity; and Cln refers to G_1 cyclins (Cln1,2) and SBF to their transcription factors (Swi4,6).

To simplify the mathematical model, we neglect the ubiquitin ligase activity of APC/C:Cdc20 (a mitotic-exit helper acting in conjunction with Cdc14). Indeed, in budding yeast, Cdc20 is dispensable, as demonstrated by viability of *cdc20Δ pds1Δ clb5Δ* triple-mutant cells.[14] Evidently, the indispensable function of Cdc20 is to degrade Securin (Pds1) and Clb5 (an S-phase cyclin); in the triple mutant, the degradation of mitotic cyclin (ClbM) is dependent solely on APC/C:Cdh1, which is activated by Cdc14. Hence, for simplicity, we consider that Cdc14 is our generic EP.

In the STAR Methods section, we provide a set of ordinary differential equations (ODEs) that describe this simplified model of the budding yeast cell cycle. Numerical integration of the ODEs (Figure 3A) presents time courses that agree reasonably well with the observed fluctuations of cell cycle regulators in wild-type budding yeast cells. Note that we do not include the size control mechanism operating at Start in daughter cells; therefore, these oscillations represent cell division cycles of mother cells. Furthermore, the SAC is not limiting in wild-type yeast cells growing under favorable conditions. Hence, this simulation of mother cell division cycles is a "limit cycle oscillator" that does not stop at the G_1 and M checkpoints (the black dots ● in Figures 1A and 3B).

Having established that the mathematical model gives a reasonable description of the yeast cell cycle, we proceed to draw bifurcation diagrams for the model (Figure 3B), using Cdh1 activity as the dynamical variable and MBF (TF) and Cdc14 (EP) as the bifurcation parameters. The red Z-shaped curve in Figure 3B is the locus of steady-state activity of Cdh1 as a function of MBF (for Cdc14 = 0) to the right and as a function of Cdc14 (for MBF = 0) to the left. The Z-shaped curve here, computed from the mathematical model, confirms the hypothetical Z-shaped curve in Figure 1A.

It is important to recognize that each half of the Z-shaped curve is itself Z-shaped in the following sense. For Cdh1 as a function of MBF (to the right in Figure 3B), the middle branch of the red curve connects to the lower branch at a negative value of MBF, as illustrated in Figure S1A. Because this connection point is at negative activity of MBF, Cdh1 cannot be reactivated as MBF activity falls to zero. This effect is crucial to the "latching" behavior of the control system. As the "gate swings open" and the cell transits from G_1 to S-G_2-M, the "spring" (the negative feedback on MBF) pulls the gate closed again and the "latch" (the stability of the metaphase steady state) holds the cell in mitosis. To leave mitosis and return to G_1, the cell must activate Cdc14 and exactly the same latching mechanism holds for this transition. As Cdc14 activity falls, Cdh1 does not inactivate because the threshold for Cdh1 inactivation occurs at a negative value of Cdc14 activity (Figure S1B). Hence, the latch catches the gate at the G_1 steady state.

On the bifurcation diagram (Figure 3B), we also plot the influence of Cdh1 activity on the steady-state levels of MBF and Cdc14. MBF becomes more active as Cdh1 activity increases (the blue curve) because Cdh1 degrades the MBF inhibitor, Nrm1.[15] In contrast, Cdc14 activity decreases with increasing Cdh1 (the orange curve) because Cdh1 degrades the Cdc14 activator, Polo.[16] We may think of these curves as "pseudo-nullclines": on the red curves, Cdh1 is at a pseudo-steady state, $d[Cdh1]/dt \approx 0$; on the blue curve, $d[MBF]/dt \approx 0$; and on the orange curve, $d[Cdc14]/dt \approx 0$. We say "≈ 0" because the actual rates of change depend on what the other variables of the dynamical system are doing at any particular time in a simulation. Wherever two pseudo-nullclines intersect is a potential steady state of the full set of ODEs; the black dot at the top, at Cdh1 = 1, is a steady state if MBF = 0 and the black dot at the bottom, at Cdh1 $\approx$ 0.0005, is a steady state if Cdc14 = 0. Taking the simulation in Figure 3A and projecting onto Figure 3B (dashed curves), we see that the pseudo-nullclines act as if they were "guiding" the temporal evolution of the dynamical system. In this case, the simulated mother-cell trajectory does not stop at the checkpoints (the black dots) because, as we have explained, the checkpoints (Start and SAC) are not operational in mother cells.

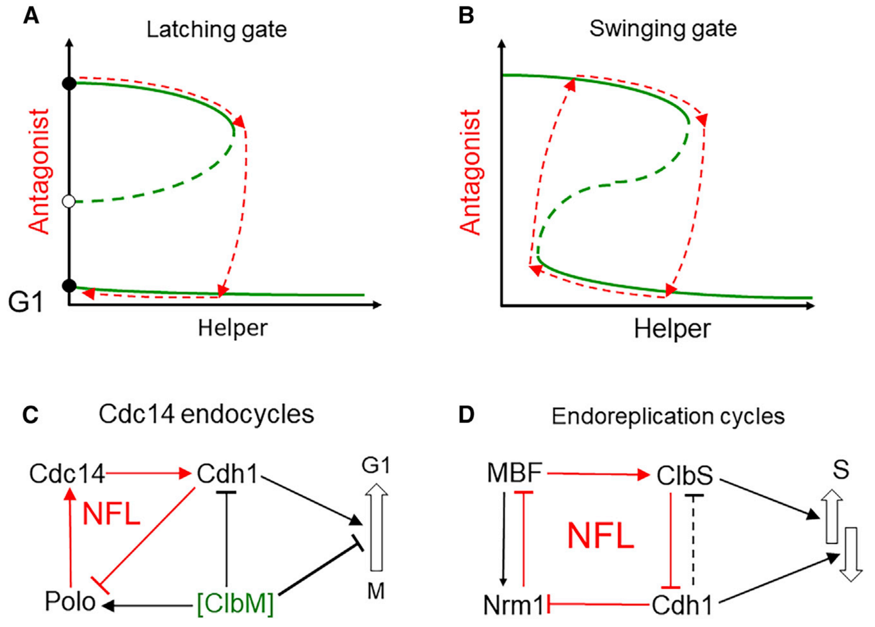

Figure 2. A latching gate, characteristic of the mitotic cycle, is converted into a swinging gate during endocycles

(A) Latching gate. Before the transition, the antagonist dominates over the activator (upper stable steady state ●). To induce the transition, a "helper" protein rises from near-zero level (red-dashed curve pointing right) and assists the activator in overcoming the antagonist (red-dashed curve pointing down). Because the helper is reliant on the antagonist, helper activity falls back to near zero (red-dashed curve pointing left) as the antagonist is inactivated.

(B) Swinging gate. A biochemical change in the control system reduces the extent of the bistable region and converts the latching gate into a swinging one.

(C) Cdc14 endocycles in the *CLB2kd*Δ strain of budding yeast. The activator of the M-to-G_1 transition is Cdh1, ClbM is the antagonist, and Cdc14 the helper. Polo kinase mediates the negative feedback loop from Cdh1 to Cdc14. In the mutant strain, ClbM activity is held constant at a characteristic mitotic level, in order to promote oscillations of Cdh1 and Polo.

(D) Endoreplication cycles in the *clb1-5*Δ strain of budding yeast. The activator is ClbS, Cdh1 is the antagonist, and the helper is MBF. We assume that Cdh1 degrades Clb6 (because in our model the antagonism of Sic1 is too weak to generate bistability). The negative feedback loop (NFL) is closed by Cdh1 degrading Nrm1, a corepressor of MBF-mediated transcription.

Cdc14 endocycles

To see how a proper latching gate (Figure 2A) in wild-type cells becomes an aberrant swinging gate (Figure 2B) in mutant cells, we first consider the case of Cdc14 endocycles (Figure 2C) in the mutant strain *CLB2kd*Δ ("KEN and D-box deleted") encoding a functional, nondegradable Clb2 (ndClbM). Degradation of mitotic cyclins is a necessary requirement for dephosphorylation of mitotic substrates by counter-acting phosphatases. In budding yeast, the bulk of mitotic dephosphorylation is catalyzed by Cdc14, which is transiently released from the nucleolus during mitotic exit.[17] In the presence of sustained activity of nondegradable Clb2, exit from mitosis is incomplete and Cdc14 release becomes periodic.[7,8]

If we express a constant low level of nondegradable ClbM in our model (e.g., ndClbM = 0.1), cell cycle progression is hardly perturbed and mitotic exit is not compromised (Figure S2A). However, at higher levels of ndClbM, the cell is unable to leave mitosis and start a new cell division cycle. For example, at ndClbM = 0.4 (Figure 4A), total ClbM > 0.4 and Sic1 < 0.02; hence, there is sufficient ClbM activity at all times to block relicensing of replication origins. Therefore, the genome cannot be replicated, although ClbS is oscillating. Nonetheless, Cdc14 is periodically inactivated and activated (sequestered in and released from nucleoli) by the negative feedback loop Polo → Cdc14 → Cdh1 –| Polo.

The existence of Cdc14 endocycles over a restricted range of expression of ndClbM is a consequence of the effect of nondegradable ClbM on the Cdh1 balance curve (Figure 4B). As we have noted, for wild-type cells (ndClbM = 0), the threshold for Cdh1 inactivation is at a negative value of Cdc14 (Figure S1B). By increasing ndClbM, the Cdh1 inactivation-threshold moves to larger values of Cdc14, eventually crossing the y axis at ndClbM ≈ 0.15. At this value of ndClbM (refer to Figure S3A), the stable steady state (●) collides with the unstable saddle point (○) underneath, and they both disappear. As a consequence, Cdh1 inactivation is no longer dependent on entering a new cell cycle and accumulating Cln and ClbS kinases; rather, the remnant ndClbM-kinase can inactivate Cdh1. In this case, there is only a single, unstable steady state of the control system, surrounded by a limit cycle oscillation (the dashed curve in Figure 4B). The limit cycle, which corresponds to the Cdc14 endocycles in Figure 4A, is independent of Cln and ClbS activities (Figure S2B). The oscillation is a consequence of the fact that as Cdc14 phosphatase activity drops, Cdh1 is inactivated by the remnant ClbM kinase activity, as evidenced by the Cdh1 inactivation-threshold at a positive value of Cdc14. It is an example of an "inhibitor-amplified negative feedback loop oscillator".[18]

Cdc14 endocycles persist over a limited range of expression of *CLB2kd*Δ, as observed in the study conducted by Lu and Cross,[7] although our range (0.15 < ndClbM < 0.66) is somewhat different from theirs. Because oscillations start where a saddle (o) and node (●) coalesce, the period of oscillation is infinitely long at first and drops significantly as ndClbM increases and the amplitude changes little (Figure S4), as observed.

Endoreplication cycles

In fission yeast cells, shutting off the synthesis of mitotic cyclin leads to periodic rounds of DNA replication in the absence of mitosis.[19] These endoreplication cycles are driven by periodic expression of fission yeast's S-phase cyclin. In budding yeast, deletion of the four mitotic cyclins (Clb1–4) causes a G_2 block because of persisting activity of the S-phase cyclin Clb5.[20] However, deletion of the *CLB5* gene supports periodic endoreplication

CellPress
OPEN ACCESS

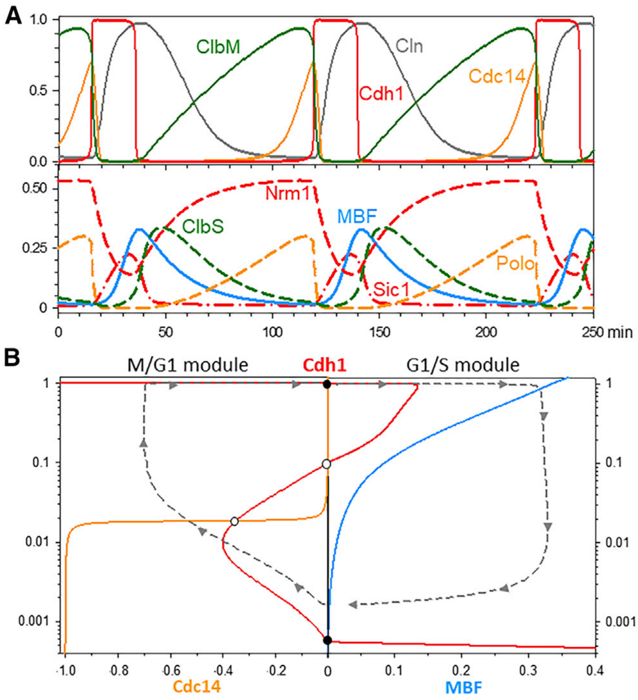

Figure 3. The cell division cycle in wild-type budding yeast viewed as a latching switch

(A) Simulated time course of a mother cell undergoing three divisions. All calculations are carried out on a system of nonlinear ODEs based on the wiring diagram in Figure 1C. The ODEs and parameter values are provided in STAR Methods and Methods S1. Upper panel: ClbM and Cdh1 are mutually antagonistic proteins, creating alternative stable states G_1 (Cdh1 active and ClbM falling rapidly) and S-G_2-M (Cdh1 inactive and ClbM rising slowly). Lower panel: behavior of the other components of the wiring diagram. By upregulating ClbS and Cln, MBF and SBF push the cell from G_1 into S-G_2-M. Polo and Cdc14 induce the reverse transition.

(B) Bifurcation diagrams. Right side: red curve, steady state activity of Cdh1 as a function of MBF activity for Cdc14 = 0; blue curve, steady state activity of MBF as a function of Cdh1. Left side: red curve, steady state of Cdh1 as a function of Cdc14 for MBF = 0; orange curve, steady state of Cdc14 as a function of Cdh1. Intersection points are steady states (●, stable; ○, unstable). The gray-dashed curves are projections of the simulated trajectories (A) onto the bifurcation diagrams.

See also Figure S1 and Table S1.

cycles in the *clb1-5Δ* strain,[6] which must be driven by an oscillation of the other S-phase cyclin, Clb6, in budding yeast (Figure 2D). Interestingly, Clb6 is the only B-type cyclin that is targeted for degradation by an SCF-dependent mechanism,[21] which evidently allows the relicensing of replication origins in the absence of Clbs 1–5.

We simulate the *clb1-5Δ* strain by reducing the synthesis of mitotic cyclins (ClbM) to zero. In this case, the cell cycle control network exhibits limit-cycle oscillations of ClbS activity driven by periodic synthesis and degradation (Figure 4C). Cln-kinase activity is maintained at a constantly high level because SBF is constitutively active in the absence of inhibition by mitotic cyclins (Clb1–4).[22]

In the absence of mitotic cyclins, the irreversible (latching) nature of the G_1-S transition is lost (Figure 4D) because the lower threshold of the Z-shaped Cdh1 balance curve, as a function of MBF, moves to a positive value of MBF activity (Figure S1A shows that the lower threshold moves into positive territory when the synthesis rate of ClbM falls below 20% of its wild-type value). Consequently, the saddle and node points coalesce (Figure S3B), the remaining steady state (at the intersection of the two balance curves in Figure 4D) becomes unstable, and the control system executes limit cycle oscillations (the dashed curve).

Other periodic events in the absence of mitotic cycles

Periodic cell cycle events uncoupled from the CDK "master" control system are observed in other circumstances. In the absence of B-type cyclin Cdk1 activity (*clb1-6Δ*) or in mutants (*cdc4* etc.) defective in degrading Sic1 (a stoichiometric inhibitor of Cdk1), cells arrest in G_1 with high Cln2-kinase activity and periodic bud formation.[23] Because constitutive Cln2-expression does not suppress this phenotype,[23] it is doubtful that periodic budding is driven by an autonomous oscillator (e.g., the small amplitude fluctuations of Cln2-kinase activity). It is more likely that the high level of Cln2-kinase activity drives continuous accumulation of bud precursors, and buds appear at regular intervals of time as bud primordia are assembled, as in the "structural model" of Fantes et al.[24] or the "dripping faucet" model of Tyson et al.[25]

Duplication of the spindle pole body (SPB) happens concomitantly with bud formation as yeast cells pass through Start. *clb1-6Δ* cells, although they bud periodically, duplicate their SPB only once in the presence of high Cln-kinase activity.[26] In contrast, endoreplicating *clb1-5Δ* cells, with oscillating activity of Clb6-kinase, overduplicate their SPBs, and overduplication becomes more pronounced in the presence of Clb5 cyclin in *clb1-4Δ* mutant cells.[26] DNA endoreplication is blocked by persistent Clb5 activity in *clb1-4Δ* cells, indicating that SPB overduplication is independent of periodic DNA replication and oscillating G_1- and S-phase Cdk1 activities. This conclusion is consistent with the observation that cells constitutively overexpressing Cln2 or Clb5 reduplicate their SPBs.[26] It is unclear whether SPB duplication in budding yeast is driven by (1) an autonomous endocycle similar to Plk4 oscillations driving centriole duplication in *Drosophila* embryos[27] or (2) a "dripping faucet" mechanism, as suggested for centrosome reduplication in S phase-arrested frog egg extracts in the presence of high cyclin E kinase activity.[28]

Persistence oscillations in energy metabolism can be detected in both budding[29] and fission yeast[30] after a block of the cell division cycle. These metabolic cycles might be driven by an autonomous oscillator in nondividing cells, but they are clearly coupled with CDK oscillations in dividing cells, presumably by Cdk1 phosphorylation of metabolic enzymes.[31,32]

As described here, periodic DNA replication and Cdc14 endocycles in budding yeast are "autonomous oscillators" only when the latching mechanism of cell cycle control fails. During normal cycles, helper proteins push the gate open, then negative feedback pulls the gate closed and the latch catches the gate at a stable steady state (either G_1 or M). In our view, endocycles are inadvertent consequences of this "logic" of cell cycle control, when the latch fails to catch.

Our view is consistent with the proposal of Manzoni et al.[8] that "events capable of repeating themselves multiple times [like Cdc14 endocycles or endoreplication cycles] are restrained to occur once per cycle by their coupling to the cyclin-Cdk engine."

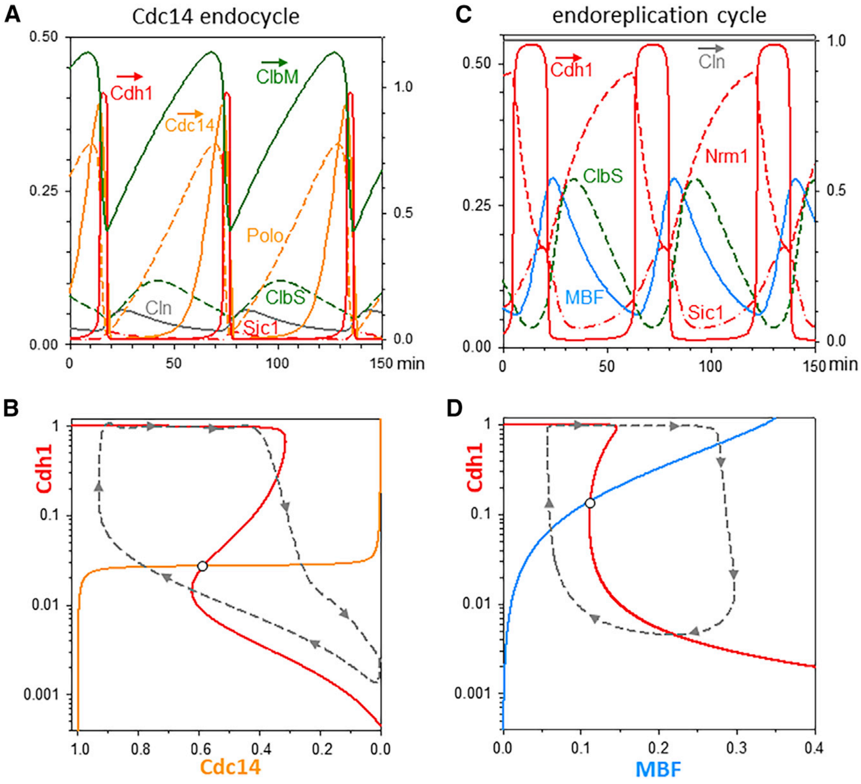

Figure 4. Endocycles

(A and B) Cdc14 endocycles in *CLB2kdΔ* cells (ndCLBM = 0.4, i.e., constant levels of nondegradable ClbM). (A) Time course simulations of the model ODEs. Note the pulsatile activation of Cdc14. ClbM activity is always high (i.e., origins of replication cannot be relicensed), and ClbS activity is always low (no DNA synthesis). Cdc14 endocycles arise from an inhibitor-amplified negative feedback loop in the presence of intermediate levels of nondegradable ClbM. (B) Pseudophase plane. Red curve, steady-state activity of Cdh1 as a function of Cdc14 (with MBF = 0); orange curve, steady-state activity of Cdc14 as a function of Cdh1. The curves intersect at an unstable steady state (○). The gray-dashed line is the limit cycle in (A) projected onto the pseudophase plane. See also Figures S2–S4 and Table S1.

(C and D) Endoreplication cycles in *clb1-5Δ* cells ($k_{sclbm}' = k_{sclbm} = 0$, i.e., no synthesis of ClbM). (C) Time course simulations. Note the periodic expression of ClbS, driving periodic rounds of DNA replication; also, because ClbM is missing, SBF is constitutively active and Cln is constantly present at a high level. Endoreplication cycles arise from an inhibitor-amplified negative feedback loop in the absence of Clbs 1–5. (D) Pseudophase plane, as in (B).

See also Figure S3 and Table S1.

It differs from the paradigm suggested by Lu and Cross[7] that "Cdc14 release, and likely other cell cycle processes, is controlled by intrinsically oscillatory modules that are entrained to a single occurrence at appropriate cell cycle positions by cyclin-Cdk cycles through a 'phase-locking' mechanism." We do not view ClbS activation and Cdc14 release as intrinsically oscillatory modules that happen to be entrained by a complementary ClbM oscillator in a phase-locked relationship but rather as inadvertent and deleterious oscillations that are normally suppressed by the CDK/Cdh1 latching-gate mechanism.

STAR★METHODS

Detailed methods are provided in the online version of this paper and include the following:

- KEY RESOURCES TABLE
- RESOURCE AVAILABILITY
  - ○ Lead contact
  - ○ Materials availability
  - ○ Data and code availability
- METHOD DETAILS
  - ○ A simple model of the budding yeast cell cycle
  - ○ Computation
  - ○ Calculation of bifurcation diagrams and pseudo-nullclines

SUPPLEMENTAL INFORMATION

Supplemental information can be found online at https://doi.org/10.1016/j.cub.2022.04.016.

ACKNOWLEDGMENTS

We acknowledge financial support from BBSRC Strategic LoLa grant BB/M00354X/1 to B.N. We thank Steven Haase for helpful advice about endocyles in budding yeast.

AUTHOR CONTRIBUTIONS

Conceptualization, B.N. and J.J.T.; software: B.N.; formal analysis, B.N. and J.J.T.; writing – original draft, J.J.T.; writing – reviewing & editing, B.N. and J.J.T.; visualization, B.N.

DECLARATION OF INTERESTS

The authors declare no competing interests.

Received: December 7, 2021
Revised: March 14, 2022
Accepted: April 7, 2022
Published: May 2, 2022

REFERENCES

1. Nasmyth, K. (1996). At the heart of the budding yeast cell cycle. Trends Genet. *12*, 405–412. https://doi.org/10.1016/0168-9525(96)10041-x.
2. Tyson, J.J., Novak, B., Chen, K., and Val, J. (1995). Checkpoints in the cell cycle from a modeler's perspective. Prog. Cell Cycle Res. *1*, 1–8. https://doi.org/10.1007/978-1-4615-1809-9_1.
3. Novak, B., Csikasz-Nagy, A., Gyorffy, B., Nasmyth, K., and Tyson, J.J. (1998). Model scenarios for evolution of the eukaryotic cell cycle. Philos. Trans. R. Soc. Lond. B Biol. Sci. *353*, 2063–2076. https://doi.org/10.1098/rstb.1998.0352.

CellPress
OPEN ACCESS

4. Novak, B., Tyson, J.J., Gyorffy, B., and Csikasz-Nagy, A. (2007). Irreversible cell-cycle transitions are due to systems-level feedback. Nat. Cell Biol. *9*, 724–728. https://doi.org/10.1038/ncb0707-724.
5. Tyson, J.J., and Novak, B. (2008). Temporal organization of the cell cycle. Curr. Biol. *18*, R759–R768. https://doi.org/10.1016/j.cub.2008.07.001.
6. Simmons Kovacs, L.A., Mayhew, M.B., Orlando, D.A., Jin, Y., Li, Q., Huang, C., Reed, S.I., Mukherjee, S., and Haase, S.B. (2012). Cyclin-dependent kinases are regulators and effectors of oscillations driven by a transcription factor network. Mol. Cell *45*, 669–679. https://doi.org/10.1016/j.molcel.2011.12.033.
7. Lu, Y., and Cross, F.R. (2010). Periodic cyclin-Cdk activity entrains an autonomous Cdc14 release oscillator. Cell *141*, 268–279. https://doi.org/10.1016/j.cell.2010.03.021.
8. Manzoni, R., Montani, F., Visintin, C., Caudron, F., Ciliberto, A., and Visintin, R. (2010). Oscillations in Cdc14 release and sequestration reveal a circuit underlying mitotic exit. J. Cell Biol. *190*, 209–222. https://doi.org/10.1083/jcb.201002026.
9. Tyson, J.J., and Novak, B. (2013). Irreversible transitions, bistability and checkpoint controls in the eukaryotic cell cycle: a systems-level understanding. In Handbook of Systems Biology, A.J.M. Walhout, M. Vidal, and J. Dekker, eds. (Academic Press), pp. 265–285.
10. Cross, F.R., Archambault, V., Miller, M., and Klovstad, M. (2002). Testing a mathematical model of the yeast cell cycle. Mol. Biol. Cell *13*, 52–70. https://doi.org/10.1091/mbc.01-05-0265.
11. López-Avilés, S., Kapuy, O., Novák, B., and Uhlmann, F. (2009). Irreversibility of mitotic exit is the consequence of systems-level feedback. Nature *459*, 592–595. https://doi.org/10.1038/nature07984.
12. Edgar, B.A., and Orr-Weaver, T.L. (2001). Endoreplication cell cycles: more for less. Cell *105*, 297–306. https://doi.org/10.1016/s0092-8674(01)00334-8.
13. Chen, K.C., Csikasz-Nagy, A., Gyorffy, B., Val, J., Novak, B., and Tyson, J.J. (2000). Kinetic analysis of a molecular model of the budding yeast cell cycle. Mol. Biol. Cell *11*, 369–391. https://doi.org/10.1091/mbc.11.1.369.
14. Shirayama, M., Tóth, A., Gálová, M., and Nasmyth, K. (1999). APC(Cdc20) promotes exit from mitosis by destroying the anaphase inhibitor Pds1 and cyclin Clb5. Nature *402*, 203–207. https://doi.org/10.1038/46080.
15. Ostapenko, D., and Solomon, M.J. (2011). Anaphase promoting complex-dependent degradation of transcriptional repressors Nrm1 and Yhp1 in *Saccharomyces cerevisiae*. Mol. Biol. Cell *22*, 2175–2184. https://doi.org/10.1091/mbc.E11-01-0031.
16. Visintin, C., Tomson, B.N., Rahal, R., Paulson, J., Cohen, M., Taunton, J., Amon, A., and Visintin, R. (2008). APC/C-Cdh1-mediated degradation of the Polo kinase Cdc5 promotes the return of Cdc14 into the nucleolus. Genes Dev. *22*, 79–90. https://doi.org/10.1101/gad.1601308.
17. Visintin, R., Hwang, E.S., and Amon, A. (1999). Cfi1 prevents premature exit from mitosis by anchoring Cdc14 phosphatase in the nucleolus. Nature *398*, 818–823. https://doi.org/10.1038/19775.
18. Novák, B., and Tyson, J.J. (2008). Design principles of biochemical oscillators. Nat. Rev. Mol. Cell Biol. *9*, 981–991. https://doi.org/10.1038/nrm2530.
19. Hayles, J., Fisher, D., Woollard, A., and Nurse, P. (1994). Temporal order of S phase and mitosis in fission yeast is determined by the state of the p34cdc2-mitotic B cyclin complex. Cell *78*, 813–822. https://doi.org/10.1016/s0092-8674(94)90542-8.
20. Dahmann, C., Diffley, J.F., and Nasmyth, K.A. (1995). S-phase-promoting cyclin-dependent kinases prevent re-replication by inhibiting the transition of replication origins to a pre-replicative state. Curr. Biol. *5*, 1257–1269. https://doi.org/10.1016/s0960-9822(95)00252-1.
21. Jackson, L.P., Reed, S.I., and Haase, S.B. (2006). Distinct mechanisms control the stability of the related S-phase cyclins Clb5 and Clb6. Mol. Cell. Biol. *26*, 2456–2466. https://doi.org/10.1128/MCB.26.6.2456-2466.2006.
22. Amon, A., Tyers, M., Futcher, B., and Nasmyth, K. (1993). Mechanisms that help the yeast cell cycle clock tick: G2 cyclins transcriptionally activate G2 cyclins and repress G1 cyclins. Cell *74*, 993–1007. https://doi.org/10.1016/0092-8674(93)90722-3.
23. Haase, S.B., and Reed, S.I. (1999). Evidence that a free-running oscillator drives G1 events in the budding yeast cell cycle. Nature *401*, 394–397. https://doi.org/10.1038/43927.
24. Fantes, P.A., Grant, W.D., Pritchard, R.H., Sudbery, P.E., and Wheals, A.E. (1975). The regulation of cell size and the control of mitosis. J. Theor. Biol. *50*, 213–244. https://doi.org/10.1016/0022-5193(75)90034-x.
25. Tyson, J.J., Alivisatos, S.G.A., Gruen, F., Pavlidis, T., Richter, O., and Schneider, F.W. (1975). Mathematical background: group report. In The Molecular Basis of *Circadian Rhythms, , J.W. Hastings, and H.-G. Schweiger, eds., pp. 85–108.
26. Haase, S.B., Winey, M., and Reed, S.I. (2001). Multi-step control of spindle pole body duplication by cyclin-dependent kinase. Nat. Cell Biol. *3*, 38–42. https://doi.org/10.1038/35050543.
27. Aydogan, M.G., Steinacker, T.L., Mofatteh, M., Wilmott, Z.M., Zhou, F.Y., Gartenmann, L., Wainman, A., Saurya, S., Novak, Z.A., Wong, S.S., et al. (2020). An autonomous oscillation times and executes centriole biogenesis. Cell *181*, 1566.e27. 1581.e27. https://doi.org/10.1016/j.cell.2020.05.018.
28. Hinchcliffe, E.H., Li, C., Thompson, E.A., Maller, J.L., and Sluder, G. (1999). Requirement of Cdk2-cyclin E activity for repeated centrosome reproduction in Xenopus egg extracts. Science *283*, 851–854. https://doi.org/10.1126/science.283.5403.851.
29. Papagiannakis, A., Niebel, B., Wit, E.C., and Heinemann, M. (2017). Autonomous metabolic oscillations robustly gate the early and late cell cycle. Mol. Cell *65*, 285–295. https://doi.org/10.1016/j.molcel.2016.11.018.
30. Novak, B., and Mitchison, J.M. (1986). Change in the rate of CO2 production in synchronous cultures of the fission yeast Schizosaccharomyces pombe: a periodic cell cycle event that persists after the DNA-division cycle has been blocked. J. Cell Sci. *86*, 191–206. https://doi.org/10.1242/jcs.86.1.191.
31. Ewald, J.C., Kuehne, A., Zamboni, N., and Skotheim, J.M. (2016). The yeast cyclin-dependent kinase routes carbon fluxes to fuel cell cycle progression. Mol. Cell *62*, 532–545. https://doi.org/10.1016/j.molcel.2016.02.017.
32. Zhao, G., Chen, Y., Carey, L., and Futcher, B. (2016). Cyclin-dependent kinase co-ordinates carbohydrate metabolism and cell cycle in *S. cerevisiae*. Mol. Cell *62*, 546–557. https://doi.org/10.1016/j.molcel.2016.04.026.
33. Ermentrout, B. (2002). Simulating, Analyzing, and Animating Dynamical Systems: A Guide to XPPAUT for Researchers and Students (Society for Industrial and Applied Mathematics).
34. Kõivomägi, M., Valk, E., Venta, R., Iofik, A., Lepiku, M., Balog, E.R., Rubin, S.M., Morgan, D.O., and Loog, M. (2011). Cascades of multisite phosphorylation control Sic1 destruction at the onset of S phase. Nature *480*, 128–131. https://doi.org/10.1038/nature10560.

STAR★METHODS

KEY RESOURCES TABLE

REAGENT or RESOURCE	SOURCE	IDENTIFIER
Software and algorithms		
XPPAUT v8.0	Ermentrout[33]	http://www.math.pitt.edu/~bard/xpp/xpp.html

RESOURCE AVAILABILITY

Lead contact

Further information about modelling should be directed to the lead contact, Bela Novak (bela.novak@bioch.ox.ac.uk).

Materials availability

This study did not generate any new reagents.

Data and code availability

The computer code is provided in Methods S1 and any additional information required to reanalyze the model reported in this paper is available from B.N. upon request.

METHOD DETAILS

A simple model of the budding yeast cell cycle

Our mathematical model provides a description of the *cdc20Δ pds1Δ clb5Δ* triple-mutant, in which Securin (Pds1), one of the S-phase cyclins (Clb5) and an APC/C activator (Cdc20) are deleted. We assume that the level of Cdc28 (Cdk1) is not limiting and the rate of Cdk1:Cyclin dimer accumulation is driven by the synthesis of different cyclins. The various Cdk1:Cyclin complexes are labelled by their cyclin component. The stoichiometric inhibitor, Sic1, binds to B-type cyclin dimers (Cdk1:Clb) to form inactive trimers. To distinguish these forms, we introduce the notation: $ClbM$ = [Cdk1:ClbM] = 'free ClbM', $ClbM_t$ = [Cdk1:ClbM] + [Cdk1:ClbM:Sic1] = 'free ClbM' + 'complex with Sic1', and similarly for $ClbS$ and $ClbS_t$. Furthermore, Clb = 'free ClbS' + 'free ClbM', and $Clb_t = ClbS_t + ClbM_t + ndClbM$, where Clb_t refers to the total Clb concentration, including non-degradable ClbM ($ndClbM$) in some simulations. To keep the model simple, we assume that Sic1 binds reversibly to both S-phase and M-phase Cdk1 complexes with the same equilibrium dissociation constant (K_{diss}). In this case, the level of inactive Sic1:Clb complexes is given by:

$$Sic1Clb = \frac{2 \cdot Sic1_t \cdot Clb_t}{B + \sqrt{B^2 - 4 \cdot Sic1_t \cdot Clb_t}}$$

where $B = Sic1_t + Clb_t + K_{diss}$. The overall Clb activity ($ClbS + ClbM$) is calculated by subtracting the inactive complexes with Sic1 ($Sic1Clb$) from total Clb (Clb_t):

$$Clb = Clb_t - Sic1Clb.$$

Since SBF is inhibited only by the mitotic Clb, $ClbM$ needs to be calculated as a fraction of Clb's not complexed by Sic1:

$$ClbM = \frac{ClbM_t + ndClbM}{Clb_t}(Clb_t - Sic1Clb)$$

The synthesis of Cln's (Cln1 & Cln2) is dependent on the SBF transcription factor, which is regulated by Cln activity (see below):

$$\frac{dCln}{dt} = k_{scln} \cdot SBF - k_{dcln} \cdot Cln$$

The synthesis of S-phase cyclin ClbS (Clb6) is driven by the MBF transcription factor, which is activated by Cln-phosphorylation and inhibited by Nrm1, a transcriptional repressor synthesized by MBF itself. The degradation of both ClbS and Nrm1 is controlled by APC/C in complex with Cdh1:

$$\frac{dClbS_t}{dt} = k_{sclbs} \cdot MBF_a - \left(k'_{dclbs} + k_{dclbs} \cdot Cdh1\right) \cdot ClbS_t$$

$$\frac{dMBF}{dt} = k'_{dis} \cdot (MBF_{tot} - MBF) - k'_{ass} \cdot MBF \cdot (Nrm1_t - (MBF_{tot} - MBF))$$

$$\frac{dNrm1_t}{dt} = k_{snrm1} \cdot MBF_a - k_{dnrm1} \cdot Cdh1 \cdot Nrm1_t$$

In these equations, $Nrm1_t = Nrm1 + Nrm1MBF$ = 'free Nrm1' + 'complex with MBF', $MBF_{tot} = MBF + Nrm1MBF$, and $MBF_a = (MBF \cdot Cln / J_{MBF} + Cln)$ = 'active MBF', i.e., the fraction of free MBF that is phosphorylated by Cln (assuming rapid phosphorylation and dephosphorylation of MBF).

The synthesis of M-phase cyclins (Clb1-4) is autocatalytic[22] and their degradation is APC/C:Cdh1-dependent:

$$\frac{dClbM_t}{dt} = k'_{sclbm} + \frac{k_{sclbm} \cdot ClbM^n}{J_{SBF}{}^n + ClbM^n} - (k'_{dclbm} + k_{dclbm} \cdot Cdh1) \cdot ClbM_t$$

The synthesis of Polo-kinase is dependent on the activity of mitotic Cdk1 complexes, and it is degraded in a Cdh1-dependent fashion:

$$\frac{dPolo}{dt} = k_{spolo} \cdot ClbM - \left(k'_{dpolo} + k_{dpolo} \cdot Cdh1\right) \cdot Polo$$

We assume that Sic1 is synthesized at a constant rate and that its degradation is controlled by dual phosphorylation.[34] The first phosphorylation step, which is assumed to be in equilibrium with dephosphorylation, is catalyzed by both Cln- and Clb-kinases, while the second step is Clb-dependent:

$$\frac{dSic1_t}{dt} = k_{ssic} - \left[k'_{dsic} + k_{dsic} \cdot Clb \cdot \frac{Cln + Clb}{J_{sic1} + Cln + Clb}\right] \cdot Sic1_t$$

The switch-like activation and inactivation of three regulatory proteins (SBF, Cdh1 and Cdc14) is described by 'Goldbeter-Koshland kinetics'. Cln-kinases phosphorylate and inactivate the inhibitor (Whi5) of the SBF transcription factor, i.e., SBF is activated by Cdk1:Cln, and SBF activity is inhibited by mitotic Cdk1 (ClbM,[22]):

$$\frac{dSBF}{dt} = \frac{(k'_{asbf} + k_{asbf} \cdot Cln) \cdot (1 - SBF)}{J_{SBF} + 1 - SBF} - \frac{k_{isbf} \cdot ClbM \cdot SBF}{J_{SBF} + SBF}$$

Cdh1 is inactivated by both S-phase and M-phase Cdk1 (recall, $Clb = ClbS + ClbM$) and reactivated by Cdc14 phosphatase:

$$\frac{dCdh1}{dt} = \frac{(k'_{acdh1} + k_{acdh1} \cdot Cdc14) \cdot (1 - Cdh1)}{J_{cdh1} + 1 - Cdh1} - \frac{(k'_{icdh1} \cdot Cln + k_{icdh1} \cdot Clb) \cdot Cdh1}{J_{cdh1} + Cdh1}$$

The nucleolar release of Cdc14 is induced by Polo-kinase:

$$\frac{dCdc14}{dt} = \frac{k_{acdc14} \cdot Polo \cdot (1 - Cdc14)}{J_{cdc14} + 1 - Cdc14} - \frac{k_{icdc14} \cdot Cdc14}{J_{cdc14} + Cdc14}$$

Computation

This set of ordinary differential equations was implemented in freely available software XPPAUT[33] in the form of an 'ode' file (see Methods S1). The dynamics of the wild-type budding yeast cell cycle were computed for the kinetic parameter values given in Table S1. Time-course simulations were performed by numerical integration of the model using the 'stiff' integrator of XPPAUT. The simulation of the endoreplication cycle was performed by setting the rate constants of ClbM synthesis (k'_{sclbm} and k_{sclbm}) to zero. The calculation of the Cdc14-endocycle was done by setting ndClbM = 0.4.

Calculation of bifurcation diagrams and pseudo-nullclines

The pseudo-nullclines were calculated using the capacity of XPPAUT to plot one-parameter bifurcation diagrams for a reduced set of ODEs. To this end, we first removed *MBF* and *Cdc14* from the system of ODEs and treated them both as parameters. To plot the Cdh1 pseudo-nullcline for the G1/S module, we set $Cdc14 = 0$ and calculated the one-parameter bifurcation diagram for $Cdh1_{ss}$ as a function of *MBF* as the bifurcation parameter. To plot the Cdh1 pseudo-nullcline for the M/G1 module, we set $MBF = 0$ and calculated the one-parameter bifurcation diagram for $Cdh1_{ss}$ as a function of *Cdc14.* For the MBF pseudo-nullcline of the G1/S module, we removed the *Cdh1* and *Cdc14* ODEs, treating them as parameters, and calculated the one-parameter bifurcation diagram for the remaining ODEs, with *MBF* as the variable and *Cdh1* as the bifurcation parameter, with $Cdc14 = 0$. The Cdc14 pseudo-nullcline of the M/G1 module was calculated in a similar fashion.

Current Biology, Volume *32*

Supplemental Information

Mitotic kinase oscillation governs the latching of cell cycle switches

Bela Novak and John J. Tyson

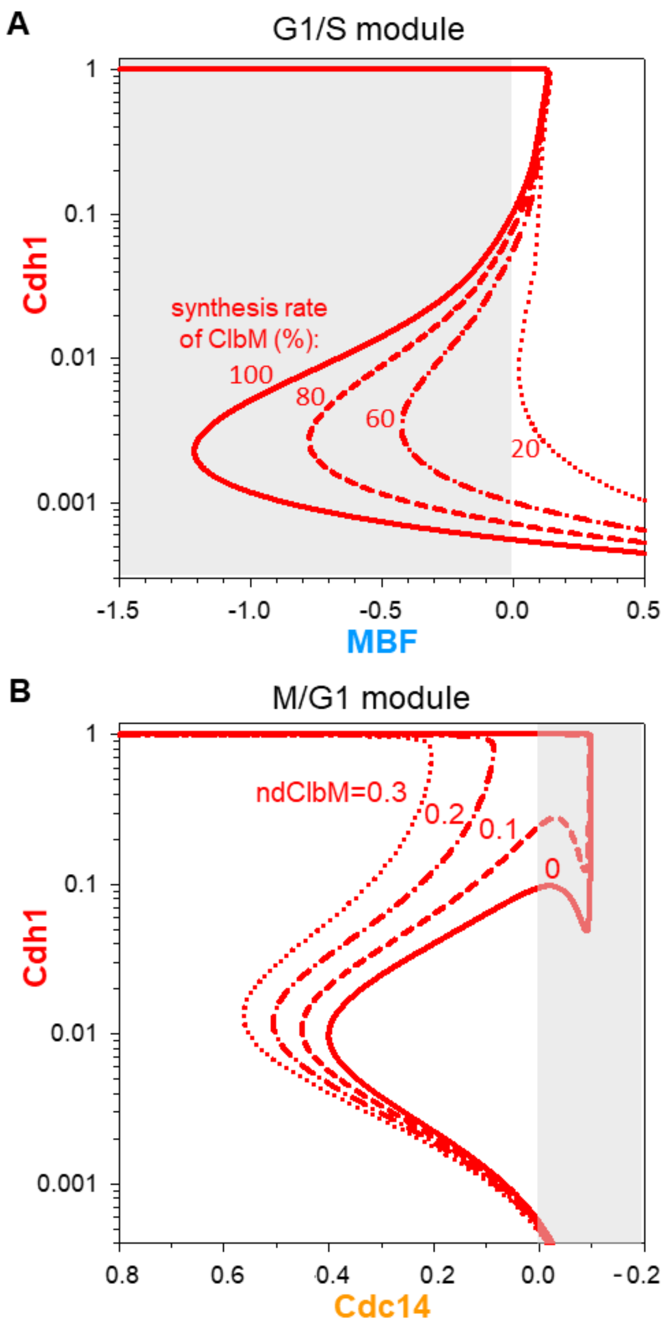

Figure S1. Bifurcation diagram for the G1/S and M/G1 modules. Related to Figure 3. (A) The steady-state level of Cdh1 as a function of MBF (when Cdc14=0) is Z-shaped, but the lower kink of the 'Z' lives in negative territory of MBF, so the switch latches at Cdh1≈0.0005 as MBF falls to zero. As the synthesis rate of ClbM decreases from its wild-type value (100%), the lower kink moves to larger values of MBF and eventually enters positive territory. Now the switch can flip back to Cdh1≈1 as MBF falls. **(B)** Similarly, the steady-state level of Cdh1 as a function of Cdc14 (when MBF=0) is Z-shaped, with the upper kink at a negative value of Cdc14. As the level of ndClbM increases from zero, the upper kink moves to larger values of Cdc14, eventually entering positive territory, and the switch fails to latch.

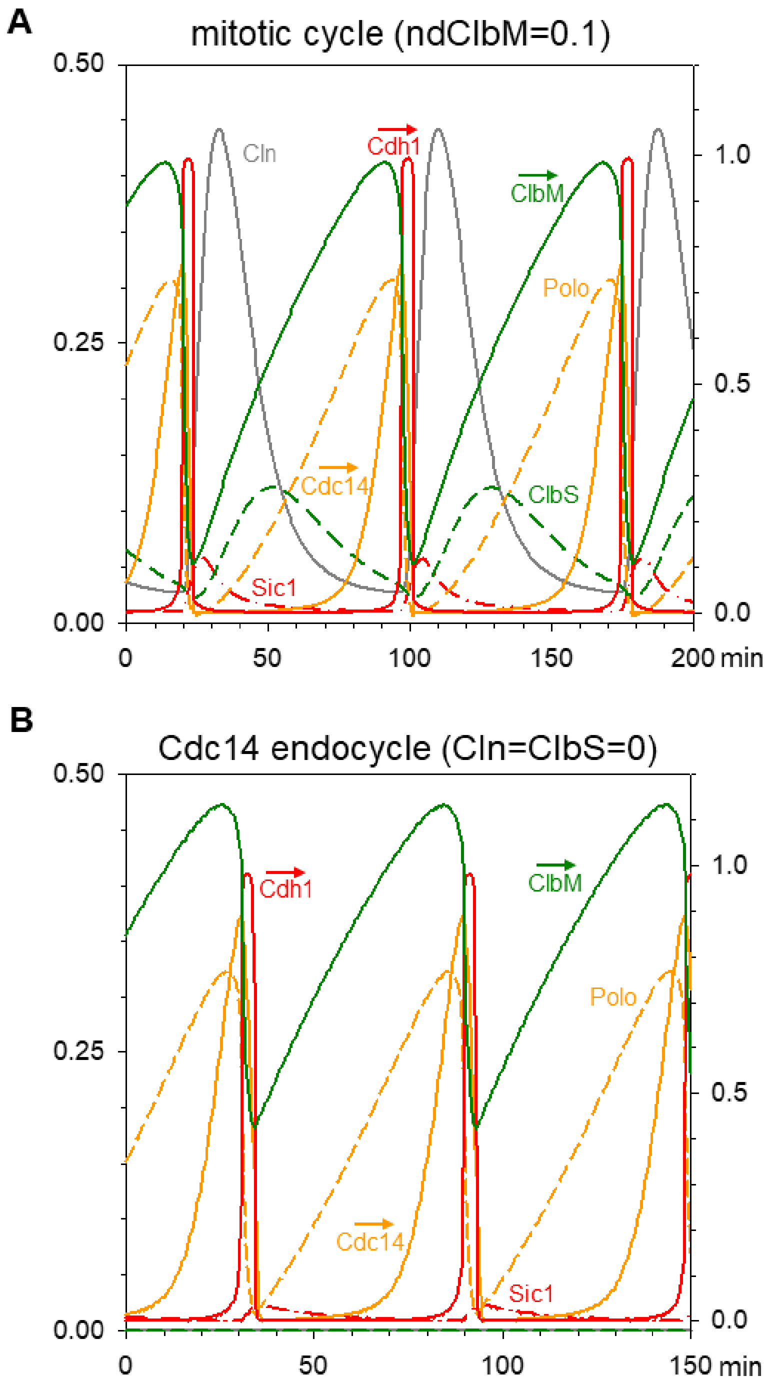

Figure S2. Properties of Cdc14 endocycles. Related to Figure 4. (A) Endocycles require a sufficiently high level of non-degradable ClbM. For ndClbM=0.1, the large post-mitotic peak of Cln, whose synthesis by SBF is very sensitive to inhibition by ClbM, indicates that ClbM activity must be very low (close to 0) as these cells exit mitosis. Indeed, the non-degradable fraction of ClbM is inhibited by the peak of Sic1 in early G_1. Low ClbM activity allows re-licensing of chromosome replication origins and the subsequent rise of ClbS activity is sufficient to initiate DNA replication and drive viable cell cycles. **(B)** Endocycles are independent of G_1- and S-phase cyclins (Cln=0 and Clbs=0).

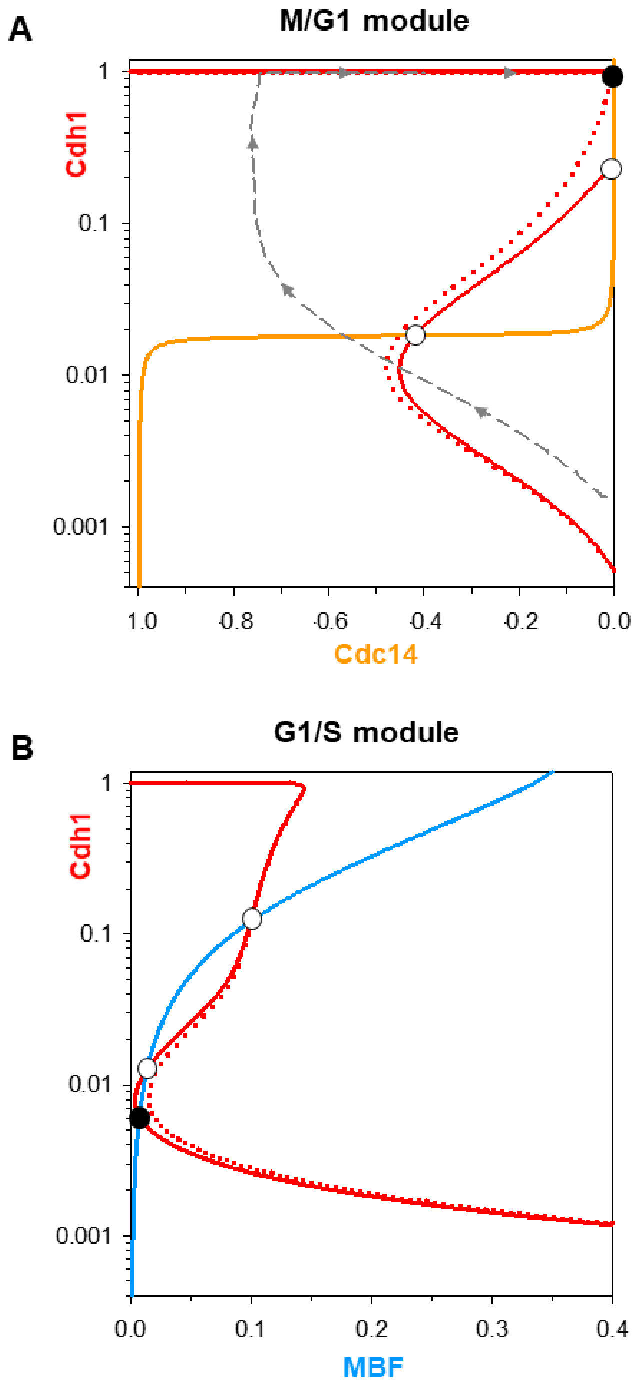

Figure S3. Endocycles arise by SNIPER ('saddle-node infinite-period') bifurcations. Related to Figure 4. Pseudo-phase plane for Cdc14 endocycles **(A)** and endoreplication cycles **(B)**, respectively. The red curves plot the locus of Cdh1 steady states for parameter values just below (solid) and just above (dotted) the SNIPER bifurcation points. As the parameter passes through the bifurcation point, a stable node (●) coalesces with an unstable saddle point (○), giving way to a limit cycle oscillation of 'infinite period'. For the solid and dotted red curves ndClbM=0.1 and 0.15 (A) and the rate of ClbM synthesis is reduced to 21% and 22.5% (B).

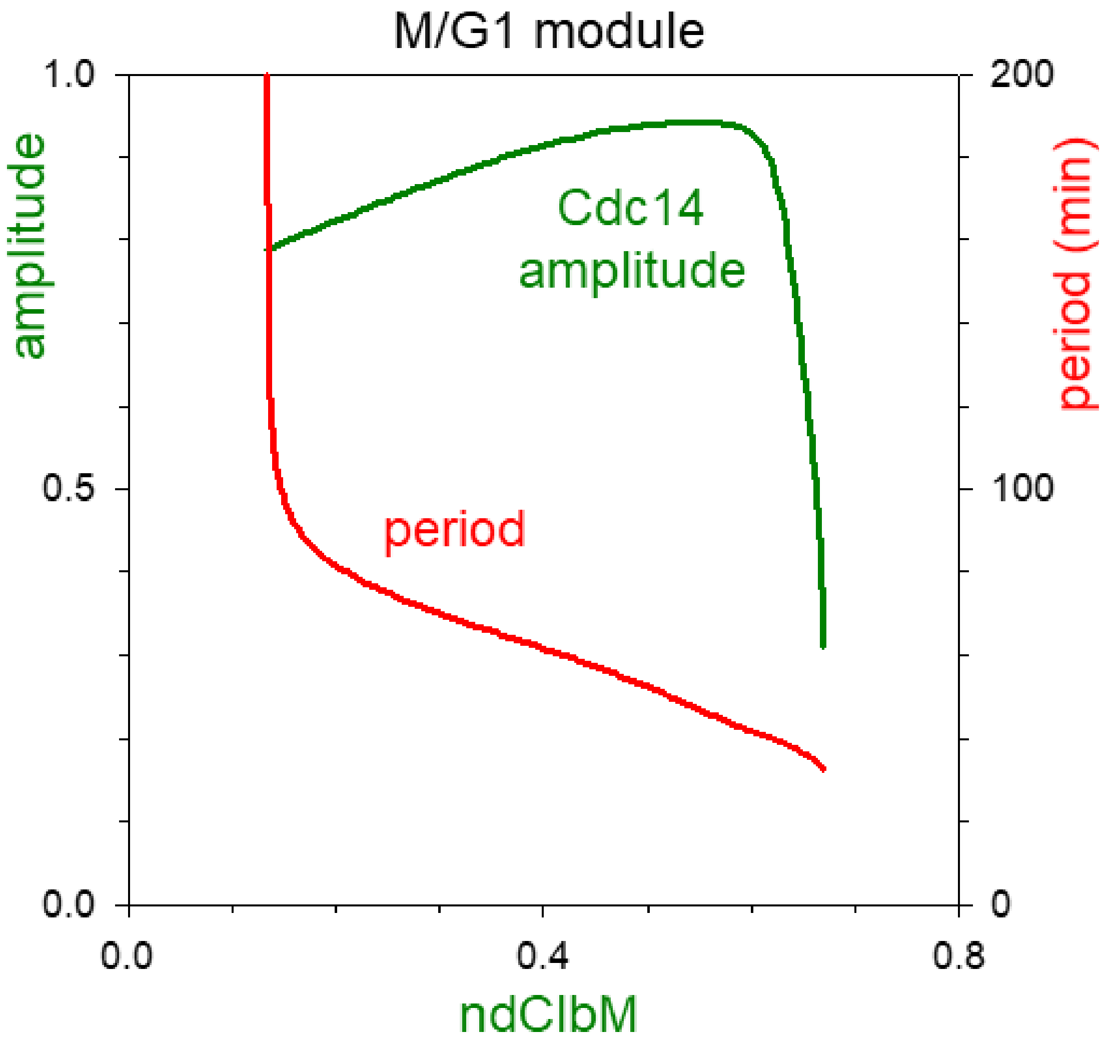

Figure S4. Bifurcation diagram for Cdc14 endocycles. Related to Figure 4. We plot the period and amplitude of the limit cycle oscillations as a function of the parameter ndClbM. Notice how the period diverges to infinity as ndClbM approaches the bifurcation point (ndClbM≈0.135).

Table S1. Parameter values of the budding yeast cell cycle model. Related to Star Methods.

**Cln synthesis/degradation & SBF regulation:**
$k_{scln} = 0.2$, $k_{dcln} = 0.2$, $k'_{asbf} = 1$, $k_{asbf} = 10$, $k_{isbf} = 25$, $J_{sbf} = 1$
**ClbS synthesis and degradation:**
$k_{sclbs} = 0.15$, $k'_{dclbs} = 0.1$, $k_{dclbs} = 0.05$
**MBF regulation by Nrm1:**
$k_{snrm1} = 0.05$, $k_{dnrm1} = 0.1$, $MBF_{tot} = 0.5$, k'_{ass}=1, k'_{diss}=0.001, $J_{mbf} = 0.01$
**ClbM synthesis and degradation:**
$k'_{sclbm} = 0.01$, $k_{sclbm} = 0.01$, $k'_{dclbm} = 0.01$, $k_{dclbm} = 1$, $J_{clbm} = 0.01$, $n = 2$
**Polo synthesis/degradation & Cdc14 regulation:**
$k_{spolo} = 0.01$, $k'_{dpolo} = 0.01$, $k_{dpolo} = 1$, $k_{acdc14} = 1$, $k_{icdc14} = 0.25$, $J_{cdc14} = 0.01$
**Sic1 synthesis/degradation & Clb binding :**
$k_{ssic} = 0.02$, $k'_{dsic} = 0.01$, $k_{dsic} = 2$, $J_{sic1} = 0.01$ $K_{diss} = 0.05$
**Cdh1 activation & inactivation:**
$k'_{acdh1} = 1$, $k_{acdh1} = 10$, $k'_{icdh1} = 0.2$, $k_{icdh1} = 10$, $J_{cdh1} = 0.01$
**Nondegradable ClbM:**
ndClbM=0 for mitotic and endoreplication cycle and ndclbM >0 for Cdc14 endocycle.
